# HSV presence in brains of individuals without dementia: the TASTY brain series

**DOI:** 10.1242/dmm.026674

**Published:** 2016-11-01

**Authors:** Jan Olsson, Hugo Lövheim, Emma Honkala, Pekka J. Karhunen, Fredrik Elgh, Eloise H. Kok

**Affiliations:** 1 Department of Clinical Microbiology, Virology, Umeå University, Umeå 90185, Sweden; 2 Department of Community Medicine and Rehabilitation, Geriatric Medicine, Umeå University, Umeå 90185, Sweden; 3 Department of Forensic Medicine, University of Tampere, Tampere 33520, Finland

**Keywords:** Herpes simplex virus, Amyloid beta aggregations, Alzheimer's disease, PCR detection, Human brain tissue, Paraffin-embedded samples

## Abstract

Herpes simplex virus (HSV) type 1 affects a majority of the population and recent evidence suggests involvement in Alzheimer's disease aetiology. We investigated the prevalence of HSV type 1 and 2 in the Tampere Autopsy Study (TASTY) brain samples using PCR and sero-positivity in plasma, and associations with Alzheimer's disease neuropathology. HSV was shown to be present in human brain tissue in 11/584 (1.9%) of samples in the TASTY cohort, of which six had Alzheimer's disease neuropathological amyloid beta (Aβ) aggregations. Additionally, serological data revealed 86% of serum samples tested were IgG-positive for HSV. In conclusion, we report epidemiological evidence of the presence of HSV in brain tissue free from encephalitis symptoms in a cohort most closely representing the general population (a minimum prevalence of 1.9%). Whereas 6/11 samples with HSV DNA in the brain tissue had Aβ aggregations, most of those with Aβ aggregations did not have HSV present in the brain tissue.

## Abstract (precis)

**Summary:** We assessed the presence of HSV types 1 and 2 in the brain tissue of a large non-institutionalised autopsy cohort, providing evidence of asymptomatic access of HSV to the brain.

## INTRODUCTION

Herpes simplex virus type 1 (HSV1) affects a majority of the population, in some cohorts up to 90% (Lövheim et al., 2015b; Smith and Robinson, 2002). The disease appears as cold sores, typically seen on the lips or face, with primary infection usually during childhood. HSV type 2 (HSV2) predominantly affects the genital area and is one of the most common sexually transmitted infections. The virus remains latent in neuronal cell bodies and reactivates due to stress, illness and other unknown factors throughout an individual's life. In some cases, individuals can develop adverse reactions such as herpes simplex encephalitis (HSE), a life-threatening disease (Whitley and Roizman, 2001).

Alzheimer's disease (AD) is the most common form of dementia and growing elderly populations will continue to put pressure on health systems to alleviate this devastating disease, which currently has no cure (Jellinger, 2006). The disease is characterised by progressive dementia, culminating in neuronal loss thought to be caused by the two main hallmarks of the disease – amyloid beta (Aβ) aggregations and neurofibrillary tangles (NFT) (Goate and Hardy, 2012).

The most common and strongest genetic risk factor for AD is the apolipoprotein E (*APOE*) epsilon 4 allele, which increases the risk of developing AD approximately threefold in heterozygotes and even more among homozygotes (Corder et al., 1995; Farrer et al., 1997; van Duijn et al., 1994). This gene can also facilitate infection with HSV (Bhattacharjee et al., 2008; Burgos et al., 2006, 2003). In addition, the rare HSE affects similar brain regions to those seen in AD (Ball, 1982). In recent years, evidence for HSV as a possible cause of AD has accumulated, and is supported by epidemiological (Letenneur et al., 2008; Lövheim et al., 2015a,b), neuropathological (Steel and Eslick, 2015; Wozniak et al., 2009), as well as *in vitro* data demonstrating AD-specific changes in neural cells following HSV infection (Santana et al., 2012).

We investigated the prevalence of HSV in 584 brain tissue samples from Tampere Autopsy Study (TASTY) cohort of 603 individuals, primarily without dementia, to assess the virus' ability to gain access to the brain without causing noticeable symptoms such as HSE. In addition, we investigated whether the presence of HSV in the brain had any effect on the presence of the neuropathology associated with AD in this cohort of community-dwelling individuals primarily free from dementia.

## RESULTS

### PCR detection of HSV brain tissue positivity

DNA was extracted from 584 paraffin-embedded brain samples from the TASTY cohort, comprising pooled middle frontal gyrus, gyrus cinguli with corpus callosum, hippocampus and cerebellum, to test for the presence of HSV genomic material. Total amount of extracted DNA for individual samples, as assessed by spectrophotometry, ranged from 1.8-57.0 µg. As the spectrophotometric value does not differentiate between intact and degraded DNA, we employed a qPCR-based assay designed to determine, in absolute numbers, each sample's content of a 41 bp target within a conserved single-copy locus in the human genome. All samples were quantified against the 41 bp target reaction and numbers of gene copies/sample were found to range from 1.2×10^5^-48.6×10^5^. Samples had similar human gene copies/reaction whether positive for the presence of HSV (*n*=11, containing 1.2×10^5^-20.2×10^5^ copies; mean 10.6×10^5^), or negative (*n*=573, containing 1.2×10^5^-48.5×10^5^ copies; mean 14.7×10^5^) (*P*=0.127)*.* All HSV1- or HSV2-positive samples were also quantified by means of a 129 bp target within the same conserved single-copy locus in the human genome, and calculating the ratio of 129/41 bp gene copies allowed for further assessment of DNA quality; as DNA fragmentation destroys longer templates at a higher rate than shorter ones, this value declines as DNA quality decreases. The 11 HSV-positive samples scored 4.5-21.0% (mean 12.6%) compared with a range of 2.4-15.5% (mean 7.8%) for 24 randomly selected negative samples (*P*=0.039).

PCR reactions directed against 62-73 bp sections of the HSV1 and HSV2 genomes were used to detect the respective viral DNA in the brain tissue samples. HSV1 PCR reactions targeted the *US5*, *UL5*, and *UL27* genes; HSV2 PCR reactions targeted *US4*, *UL5* and *UL29*. In the trade-off between maximal sensitivity on the one hand and high standards of specificity and reproducibility on the other, no Ct cut-off value was employed, and only samples positive for at least two of the PCR reactions were scored positive. The independent PCR reactions thereby confirm each other (Mahony et al., 1993). Of 584 analysed samples, 10 were positive for HSV1, whereas one was positive for HSV2 (positivity in *US4* and *UL29*, but not *UL5* PCR reactions). None were positive for both HSV1 and HSV2.

In detecting HSV1 DNA, the *US5*, *UL5* and *UL27* PCR reactions were positive in 18 (3.1%), 16 (2.7%) and 19 (3.3%) out of 584 analysed cases, respectively. There were 27 cases (4.6%) with only one positive reaction, and 10 (1.7%) where at least two reactions were positive. Six out of these 10 were triple-positive. Mean Ct values were 38.0±2.7 (±s.d.) for cases with at least two positive reactions compared with 39.4±1.0 for those with only one positive reaction (*P*=0.156). These Ct values are high, indicating sub-optimal qPCR conditions, probably due to the presence of inhibitory substances. However, agarose-gel electrophoresis staining showed unambiguous correlation between a positive qPCR score and the presence of a product band of correct size (data not shown). The correlations between positivity of the different PCR reactions were 0.395 (*US5* to *UL5*), 0.383 (*UL5* to *UL27*) and 0.414 (*UL5* to *UL27*), respectively (*P<*0.001 for all correlations). In the following analyses, the HSV1 and HSV2 PCR data are pooled (see Table 1 for HSV DNA-positive case characteristics).

**Table 1.**
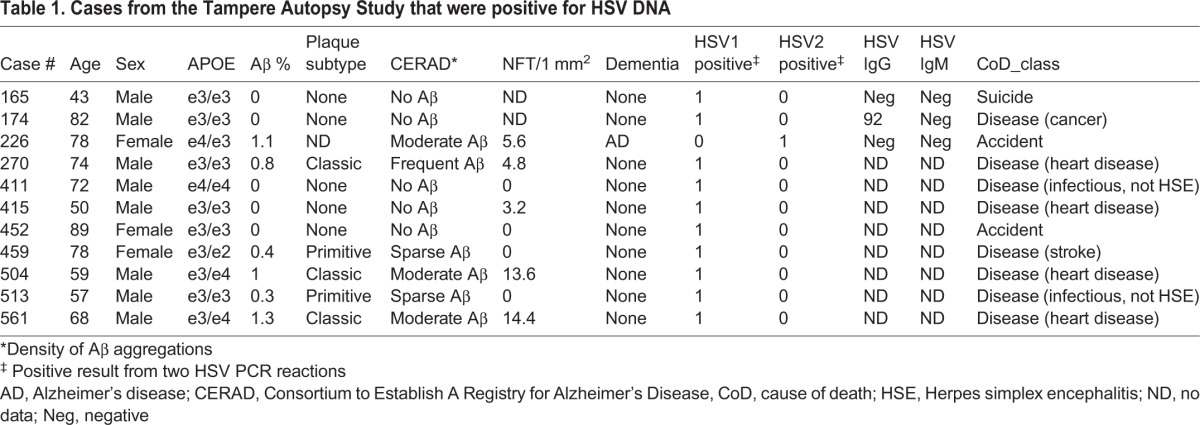
**Cases from the Tampere Autopsy Study that were positive for HSV DNA**

### HSV brain tissue positivity

**Fig. 1.**
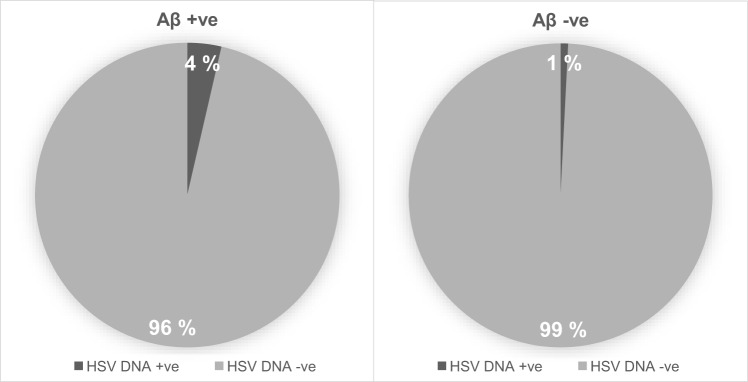
**The prevalence** **of HSV DNA positivity in cases separated by Aβ pathology presence.** Cases with HSV DNA present are depicted in dark grey, versus those without HSV DNA, split into groups with and without Aβ positivity.

Cases (*n*=10 HSV1 and 1 HSV2, total of *n*=11 HSV-positive) who had at least two positive PCR reactions were regarded as HSV DNA-positive in brain tissue (11/584=1.9%). Age, sex and *APOEε4* carriership (*P*=0.413, *P*=0.754 and *P*=0.746, respectively) did not affect the risk of being HSV DNA-positive. Individuals were aged 60 years and over in 59% of the cases (*n*=346), of which 2.0% (7/346) had a prevalence of HSV positivity in brain tissue. The proportion of cases with Aβ aggregations (Fig. 1) was 6/11 (54.5%) among those positive for HSV DNA in brain tissue compared with 160/530 (30.2%) among those negative for HSV DNA (Fisher's exact test, *P*=0.101 of cases with available Aβ aggregation data), and for NFT, 5/9 (55.6%) compared with 194/465 (41.7%) (Fisher's exact test, *P*=0.502 of cases with NFT data). The proportion of cases with both Aβ aggregations and NFT was 4/9 (44.4%) among HSV DNA-positive cases compared with 81/451 (18.0%) (Fisher's exact test, *P*=0.097 of cases with complete Aβ aggregation and NFT neuropathology data). Six cases of the TASTY cohort had been diagnosed with AD while alive, of which one individual was positive for HSV DNA in brain tissue. The proportion of individuals with HSV DNA positivity was 1/6 (16.7%) among those with AD, compared with 10/551 (1.8%) of non-demented cases (Fisher's exact test, *P*=0.113 of cases with documented dementia information).

### Serology

It was possible to obtain serology data for 141 of the cases (24.1%), of which 121 (85.8%) were positive for anti-HSV IgG. Mean age was 57.6±15.3 for anti-HSV-IgG-negative cases and 64.5±17.5 for those anti-HSV-IgG-positive (*P*=0.103). Sex and *APOEε4* carriership did not impact on the risk of carrying HSV; 74/88 (84.1%) of the men and 47/53 (88.7%) of the women were anti-HSV-positive (*P*=0.449), and 45/53 (84.9%) of the *APOEε4* carriers compared with 76/88 (86.4%) of those not carrying an *APOEε4* allele (*P*=0.810).

### Serology and neuropathology

Among anti-HSV-IgG-positive cases for which Aβ aggregation data was available, 36/111 (32.4%) were Aβ-aggregation-positive compared with 2/19 (10.5%) of those anti-HSV-IgG-negative [χ^2^: *P*=0.052; adjusted for age in a binary logistic regression: odds ratio (OR) 2.771, 95% confidence interval (CI) 0.567–13.541, *P*=0.208]. NFT was seen among 30/77 (39.0% of anti-HSV-IgG-positive) compared with 3/12 (25.0% of anti-HSV-IgG-negative) with NFT neuropathology data in this cohort (χ^2^: *P*=0.352; age-adjusted: OR 1.264, CI 0.281–5.681, *P*=0.760).

### IgM and IgG serology

There were 15/141 (2.5%) anti-HSV-IgM-positive. All but one of them were also anti-HSV-IgG-positive and hence regarded as reactivated infections. One 58-year-old male was IgM-positive and IgG-negative, which indicates primary infection. The individual had no Aβ aggregations or NFT. Among those positive for anti-HSV IgG, those who were also positive for anti-HSV IgM were younger (55.3±16.7 versus 65.6±17.3, *P*=0.038) and more often male [12/74 (16.2%) versus 2/47 (4.3%), *P*=0.045]. Anti-HSV IgM antibodies were equally common among those with or without an *APOEε4* allele [6/45 (13.3%) versus 8/76 (10.5%), *P*=0.641]. Adjusted for age and sex, there were no significant relations between anti-HSV IgM and Aβ aggregations or NFT (*P*=0.346 and *P*=0.519, respectively).

### Serological levels and correlations

Anti-HSV IgG level (among those anti-HSV-IgG-positive) was affected by age and sex of the cases (multiple linear regression: age β=0.298, *P*=0.018, male sex β=11.563, *P*=0.011). The level was 81.8±23.0 among those who carried at least one *APOEε4* allele compared with 74.2±22.6 among those who did not (*P*=0.079). There were no significant correlations between anti-HSV IgG level and Aβ-immunoreactivity (IR) percentage and NFT count (Pearson correlation −0.002, *P*=0.987 and –0.039, *P*=0.734, respectively).

### Serology and PCR positivity

Blood plasma was available for only three of the HSV-DNA-positive samples. Interestingly, two out of these three cases – one HSV1-positive and the single HSV2-positive – were negative for anti-HSV IgG, compared with 120/138 (87.0%) of those HSV-DNA-negative with available serological data (Fisher's exact test *P*=0.053).

## DISCUSSION

**Fig. 2.**
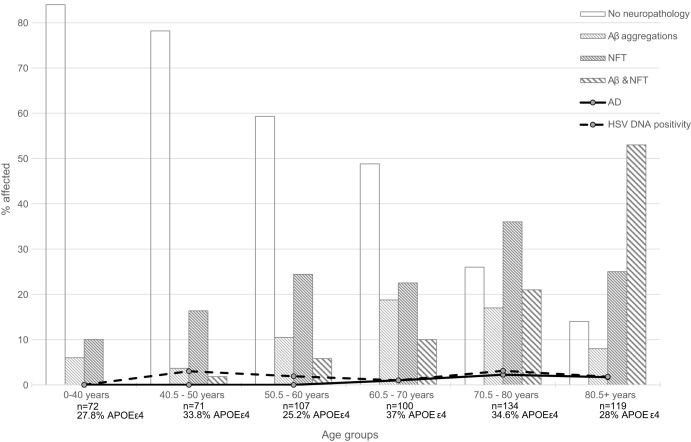
**TASTY cohort characteristics.** Changes in age distribution of HSV DNA presence in brain tissue, *APOEε4* carriership, Aβ aggregations, NFT, and combined neuropathology, as well as Alzheimer's disease cases of the TASTY cohort. Please note that only those cases with neuropathology data for both Aβ aggregations and NFT were included in the figure.

Our main finding was that in an unhospitalised cohort (see Fig. 2) of 603 individuals primarily without dementia, we detected HSV in brain tissue of 11 (1.9%) of the 584 cases from which it was possible to extract DNA. Our serological analyses of 141 cases revealed 85.8% carrying HSV, which is slightly lower than – although in line with – previous epidemiological studies of the virus' presence (Lövheim et al., 2015a,b; Smith and Robinson, 2002).

We used strict criteria for positivity, possibly underestimating the actual number because of sample degradation. Preparations from FFPE brain tissue generally yield more severely degraded DNA than preparations from, for example, fresh frozen tissue (Ferrer et al., 2007). In addition, formalin storage length has been suggested to affect genomic DNA yield (Wang et al., 2013; Ferrer et al., 2007), in which the current samples were fixed for around 2 weeks (Kok et al., 2009). We assessed the quality of prepared human DNA as an indicator of chances for successful HSV DNA detection, assuming that DNA degradation affects human and viral DNA equally. In fact, samples positive for HSV DNA had a slightly higher average 129/41 bp human DNA ratio than those negative for HSV DNA, possibly indicating that some samples are false negatives. The indicated 1.9% of HSV-positive samples should therefore be regarded as a minimum estimate, especially in light of earlier studies that have focussed on AD patients in comparison with non-AD controls. Such studies have reported a HSV prevalence ranging from 21.7-100% for non-AD individuals (Fraser et al., 1981; Bertrand et al., 1993; Baringer and Pisani, 1994; Itzhaki et al., 1997; Jamieson et al., 1991, 1992; Wozniak et al., 2005), although they have generally dealt with older individuals and substantially smaller sample sizes. Sequence variations of the infecting HSV strains might also have resulted in missed infections. It should also be noted that the cohort stems from a Finnish population not previously explored for HSV DNA.

To the authors' knowledge, a study of this size has not previously been performed, and it sheds light on the prevalence of HSV in brain tissue in a unique cohort most closely representing the general population, including individuals of all ages. The results suggest HSV presence in brain tissue is not unusual, and further indicates the existence of persistent or latent infections within the central nervous system (CNS) without producing encephalitis symptoms. The results also indicate that spread to the CNS is much more common for HSV1 than for HSV2, consistent with a low proportion of HSV2 being found in human brain in other studies (Lin et al., 2002).

The small number of HSV-DNA-positive samples resulted in a lack of power in most analyses. Although interpretation must be made with appropriate caution, some non-significant results might still be worth commenting on. The proportion having Aβ aggregations among those who had HSV DNA in brain tissue (i.e. tested positive in two or more PCR analyses) were 6/11 (54.5%) compared with 30.2% among those who were HSV-DNA-negative (*P*=0.101), 16.7% of those with AD had HSV DNA present in brain tissue compared with 1.8% of non-demented cases (*P*=0.113), and anti-HSV sero-positivity was close to being significantly associated with an increased risk of Aβ aggregations (*P*=0.052). These results are not in disagreement with previous studies suggesting that Aβ aggregations and/or AD are associated with HSV (Ball, 1982; Itzhaki, 2014; Itzhaki et al., 2016; Letenneur et al., 2008; Lövheim et al., 2015a,b; Steel and Eslick, 2015), but should be confirmed in larger studies. However, most of the individuals that had Aβ aggregations did not have detectable HSV DNA in brain tissue. Although this might in part be explained by the sensitivity of our analyses, recent studies are pointing towards the Aβ reaction being a part of the innate immune system (Itzhaki et al., 2016; Kumar et al., 2016), with a broad antimicrobial effect against bacteria, fungi and viruses including HSV (Bourgade et al., 2015; Soscia et al., 2010; White et al., 2014). Several bacteria and viruses have been demonstrated as being able to trigger the Aβ reaction in cultured cells and mice (Kristen et al., 2015; Little et al., 2014; Miklossy et al., 2006; Santana et al., 2012; Shipley et al., 2005). A possible explanation might therefore be that previous infections leave scars in the form of Aβ aggregations; however, certain persistent infections including HSV might continue to trigger the Aβ production for prolonged periods of time to produce accumulation of Aβ and ultimately Alzheimer's disease. A number of recent studies have leant weight to support the theory of a pathogen-mediated AD hypothesis (Bourgade et al., 2016; Miklossy, 2011). These include investigations into fungi (Pisa et al., 2015), bacteria (Miklossy, 2015; Potgieter et al., 2015) and even their by-products (Kell and Pretorius, 2015), and point towards a potential role of several infectious pathogens in AD neurodegeneration.

Two out of three individuals positive for HSV DNA in brain tissue with sera available were HSV sero-negative, a borderline significantly lower proportion (*P*=0.053) compared with those negative for HSV DNA, among whom 87.0% were HSV sero-positive. Although this unexpected result must be interpreted with caution, one possible explanation could be individuals that for some reason have not developed a proper immune response to HSV infection (here presented as low levels of specific antibodies), might have an increased risk of HSV spread to the CNS. Alternatively, sample degradation and interference with the ELISA system resulting from post-mortem-altered blood samples could be a source of error; however, the high prevalence of HSV sero-positivity among those negative for HSV DNA in brain tissue indicates a sufficient sensitivity in the ELISA analyses also for post-mortem samples.

In conclusion, we report epidemiological evidence of HSV presence in brain tissue from individuals who did not show encephalitis symptoms in a cohort most closely representing the general population (prevalence 1.9%). Six out of 11 with HSV DNA in brain tissue had Aβ aggregations, although most of those with Aβ aggregations did not have HSV present in brain tissue.

## MATERIALS AND METHODS

### Cohort and neuropathology

The TASTY cohort has been described in detail elsewhere, including Aβ aggregation and neurofibrillary tangle (NFT) data acquisition (Kok et al., 2009). Briefly, 603 individuals (388 males, 215 females; 64% male) aged 0-97 years (average 63 years) underwent autopsy at the Department of Forensic Medicine, University of Tampere, Finland from 2002-2004 (see Table 2). Samples underwent sectioning and staining with Bielschowsky silver stain according to standardised procedures, and were measured as positive for Aβ aggregations (*n*=541, 92.6% of the TASTY cohort were tested; *n*=166, 30.7% were positive) and Aβ-IR (immunoreactivity) as a percentage of area covered by Aβ aggregations (average Aβ-IR was 0.45%, range 0-5.4%), and neurofibrillary tangle (NFT) data (data available for *n*=474, 81.2%; *n*=199, 42.0% positive; NFT/1 mm^2^ average=3.4, range 0-60.8) (Kok et al., 2009). Of the 603 cases in the TASTY cohort, 31.1% were *APOEε4* carriers (*n*=187), 584 had brain tissue blocks and 141 had serology samples available for the current study. Not all cases had all data available owing to unavailable or missing samples, most often caused by cause of death hampering sample collection.

**Table 2.**
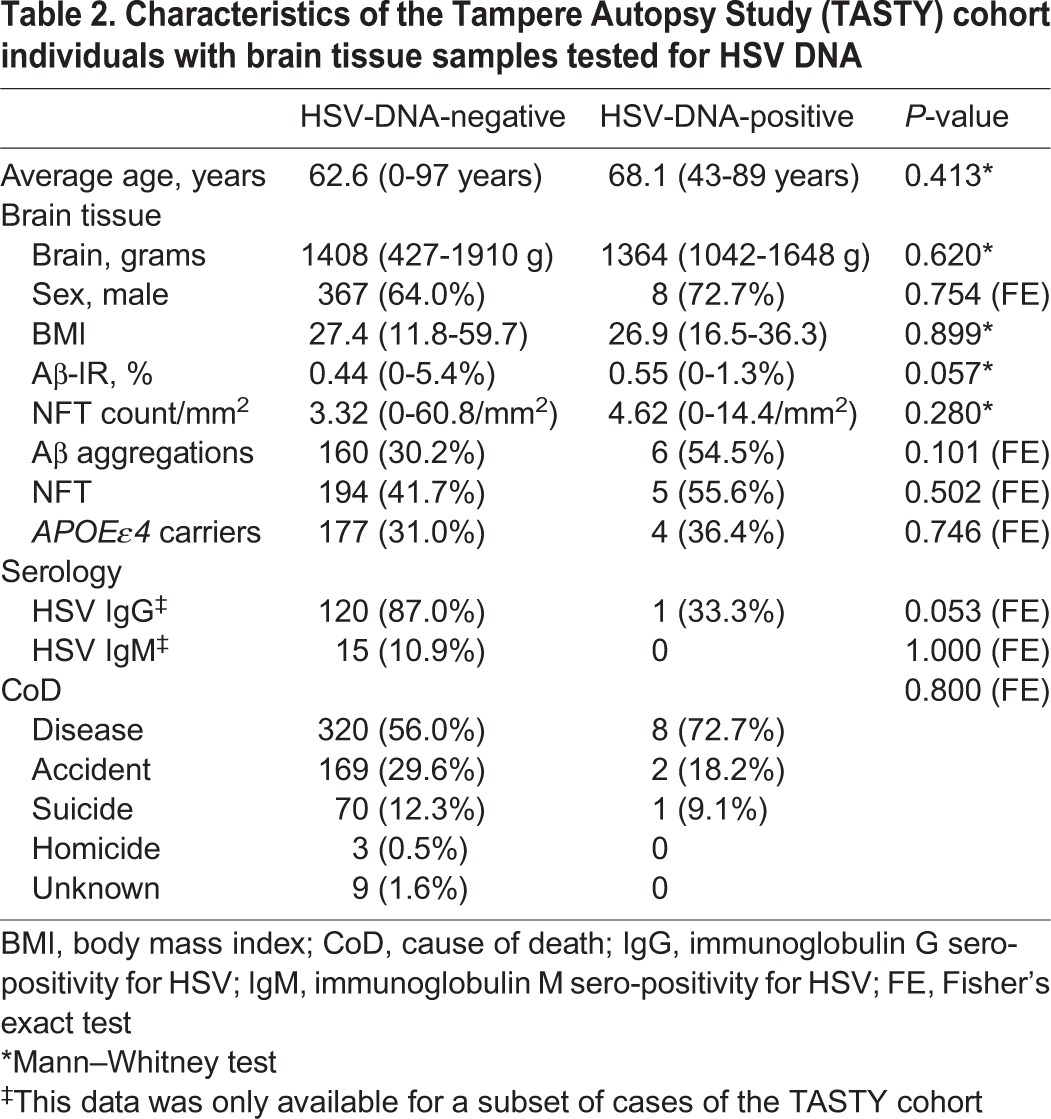
**Characteristics of the Tampere Autopsy Study (TASTY) cohort individuals with brain tissue samples tested for HSV DNA**

The autopsy series and its use were approved by the National Authority for Medicolegal Affairs in Finland (1239/32/200/01). The Regional Ethical Review Board in Umeå, Sweden approved the study (2013/277-31).

### DNA extraction

Paraffin-embedded tissue blocks of four brain regions (middle frontal gyrus, gyrus cingula with corpus callosum, hippocampus and cerebellum) were sampled 4-6 times with 20-µm-thick sections and transferred into sterile microfuge tubes. Tissue was deparaffinised according to the guidelines outlined by Greer et al. (1994). Briefly, 1 ml xylene was added to each tube, centrifuged at 16,000 ***g*** for 5 min, after which the supernatant was discarded. This procedure was carried out twice. The xylene was then removed in three washes with 99% ethanol with centrifugation at 16,000 ***g*** for 5 min in each washing step. Tubes were left open in a dust-protected cabinet for one hour to allow the remaining ethanol to evaporate. Thereafter, DNA was extracted with a LIAISON Ixt pipetting/extraction instrument fitted with a LIAISON Ixt DNA Extraction kit (Diasorin). DNA Pretreatment Buffer 2 supplemented with Proteinase K (200 µl+10 µl was added to each sample, and the tubes were incubated at 56°C overnight. Samples were then centrifuged (10 min, 20,000*** g***) to sediment residual material. DNA was prepared from the digested samples and eluted into 50 µl elution buffer.

### Quality assessment of extracted DNA

The total amount and purity of DNA for each sample was assessed by spectrophotometry (NanoDrop 1000 Full Spectrum UV/Vis Spectrophotometer, Wilmington, USA). The total amount of DNA was obtained in ng/μl, and the A260/280 ratio was calculated as an indicator of protein impurities. In order to assess DNA quality with respect to fragmentation, samples were subjected to a qPCR-based quantification of 41 bp and 129 bp targets within a conserved single-copy locus in the human genome using a KAPA Human Genomic DNA Quantification and QC Kit (KAPA Biosystems, Cape Town, South Africa). Samples were diluted 1:100 in 10 mM Tris pH 8.0+0.05% Tween 20 (DNA dilution buffer) to fall within the dynamic range of the assay, and the qPCR reaction was performed according to the manual for the product on an ABI StepOne Plus instrument. Absolute quantification of 41 bp and 129 bp targets of human genomes in samples was achieved using the standard provided in the kit.

### PCR methods

PCR reactions directed against conserved regions of the HSV1 and HSV2 genomes were used to detect the respective viral DNA in the samples. Primers and TaqMan probes were designed with the online software Primer3Plus (Untergasser et al., 2007). Crucial design parameters were: T_m_ for primers 60°C, T_m_ for probes 70°C, length of amplified regions 60-75 bp, and no sequence match to other reference genomes. Primers and probes were ordered from EurofinsGenomics, Ebersberg, Germany, with the sequence of primers and probes, and the size of amplicons described in Table 3. DNA prepared from in-house HSV1 or HSV2 cultivations were used as positive controls. DNA was diluted 1:10, after which 10 µl was added to a final volume of 25 µl PCR mix containing TAQMAN UNIV Master MIX (Life Technologies, Carlsbad, CA, USA), 0.3 µM of each primer and 0.2 µM probe. Amplification reactions included an initial 15-min denaturation step at 95°C, followed by 43 cycles of 15 s at 94°C, and 60 s at 60°C. The reactions were performed on an ABI 7900HT instrument, and results were analysed with the SDS software set to automatic baseline and threshold (Automatic Ct).

**Table 3.**
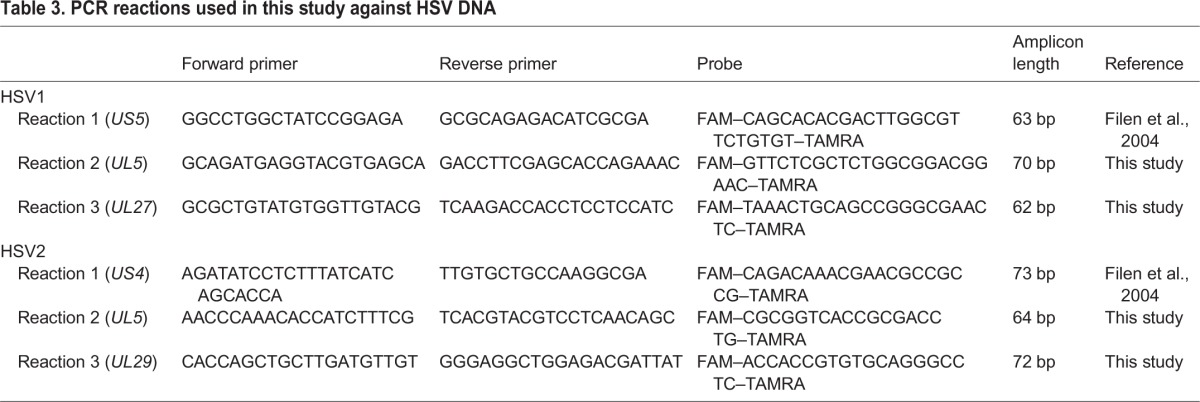
**PCR reactions used in this study against HSV DNA**

### Serology

Plasma collected at autopsy was stored at −80°C. Samples were thawed, cleared of particular matters by centrifugation (10 min, 20,000 ***g***), and analysed for anti-HSV IgG and IgM antibodies using ELISA as described previously (Lövheim et al., 2015a), with the exception that a new HSV1 isolate (Umeå clinical isolate 3458-13) has been introduced for antigen production. The IgG antibody activity of the individual samples was expressed in arbitrary units (AU), achieved by dividing the sample's absorbance with that of a positive reference sample, and multiplied by 100. Samples with IgG values of 5 AU or above were regarded positive for HSV IgG antibody content. IgM ELISA results were dichotomously determined positive or negative with the cut-off value set at net absorbance ≥0.15.

### Statistics

SPSS for Windows (version 23; IBM) was utilised in statistical analyses. Variables used were Aβ aggregations (present yes/no; Aβ-IR %), NFT (yes/no, NFT count), *APOEε4* allele carriership (yes/no), sex, HSV DNA positivity (two positive tests/no), anti-HSV IgG (present yes/no; AU), and anti-HSV IgM (present yes/no).
